# Book Review: Clés des songes et sciences des rêves. De l'Antiquité à Freud

**DOI:** 10.3389/fpsyg.2016.01172

**Published:** 2016-08-15

**Authors:** Giorgia Morgese

**Affiliations:** Department of Dynamic and Clinical Psychology, Sapienza University of RomeRome, Italy

**Keywords:** Clés des songes, sciences des rêves, oneirocritica, interpretation of dream

*Clés des songes et sciences des rêves* edited by Jacqueline Carroy and Juliette Lancel, explores the art of interpreting dreams from Ancient times to Freud.

The aim of this paper is to analyze the western tradition of studying oneirocriticism: the book of interpretation of dreams, *clés des songes*.

The authors have chosen to use the “longue durée” approach in their study of the history of dream interpretation (“longue durée” is an expression used by the French Annales School of historical writing to designate their approach to the study of history, which gives priority to long-term historical structures over events). This approach involves analyzing human dreams from case studies according to the intellectual, social, and historical contexts. Carroy and Lancel follow the same approach used by Pick and Roper's (2004) book, but with a focus on what they called “genre littéraire d'écrit et de pratique bien particulier” (a very particular kind of literary writing and practice) in Western history. So the book is probably one of the finest books dedicated to the history of *clés des songes* putting emphasis on German and French context.

The book starts with a focus on how the first treatise devoted to oneirocritica, written by Artemidorus, led to an important anachronistic paradox: the oneirocritica differentiates itself from false divination, as well as from magical or religious approaches. In fact, during Ancient times, dreams were regarded as messages from the gods to men, giving them a supernatural significance. Vande Kemp (1981) refers to this as the transpersonal period. On the contrary Artemidorus, as Julien du Bouchet shows in the second chapter, did not particularly emphasize the role of the gods in dreams, and appears as a pragmatic practitioner to illustrate tools and skills for interpreting dreams. According to the authors, this approach led to the survival and spread of *clés des songes* from Ancient times up to now.

This book, with the contributions of expert researchers in this field, promotes the *clés des songes* as a social and historical concept. In the French context, the words “songe” and “rêve” became synonymous between the seventeenth and eighteenth centuries. After the Eighteenth century, scholars used the word “rêve” to mean an internal physiological or psychological phenomena. It was only after the nineteenth century that the *clés des songes* was taken into account, thanks to the French translations of many books on the topic. The history of “longue durée” proposed in this book, underlines how the historical trajectory is far from linear.

In the introduction, the authors highlight the work of Aristotle as the two different representative points of view of the eighteenth and nineteenth centuries: dreams as a natural phenomena and dreams as a sign or as having premonitory meaning. However, Freud changed this perspective with the introduction of psychoanalysis.

In regards to interpreting dreams as signs or as having premonitory meaning, Julien du Bouchet introduces Artemidorus of Daldi, who wrote the *Oneirocritica*, the first treatise about *cle de songes* written in the second century AD. The title of this treatise literally means *the interpretation of dreams*.

The following chapters deal with *clés des songes* in the Byzantine context and during the Middle Ages. Andrei Timotin highlights the aim of the Byzantine oneirocritica: to christianize the pagan discipline illustrated in Artemidorus' treatise (third chapter). The Byzantine oneirocritica did not deal with the method of interpretation, but rather, it dealt with the hermeneutic procedures: the use of the analogy, the antinomy, the word pun, the use of writing and deductive reasoning.

Thanks to Jean-Claude Schmitt, we also know the role of *clés des songes* during the Middle Ages, the period that introduced the art of dream interpretation into Europe (fourth chapter). During the Middle Ages, this technique was also spread in the popular culture of modern Europe linked to diffusion of horoscopes.

In the fifth and sixth chapters, the authors, Claire Gantet and Juliette Lancel, take into account the *clés des songes* in the German and French contexts. The first German translation of Artemidorus' treatise was published in 1540 by the apothecary Hermann Ryff (1500–1548). From 1551, Martin Luther's followers replied with more than 20 translations, but from 1814, the *clés des songes* changed its perspective, promoting a new road in dream interpretations, taking into account the role of the soul.

Juliette Lancel introduces how the *clés des songes* arrived in France. The Muslim oneiromancy was known in French thanks to physician Pierre Vattier's translation in 1664. Lancel highlights the fact that Vattier is a physician and tries to find the reasons why one physician would have translated an Arabic *clé de songes*. His work introduces the problem of the opposition of biblical authority.

On the other hand, Vincent Barras provides an overview of the pioneers of the “science du rêve” who regarded dreams as a natural phenomena and developed diverse theories ranging from the physiological to the semiological. Thanks to these dream theories, the process of the secularization of dreams began in the Western tradition (Carroy et al., 2006; Carroy, 2012). The physiological perspective in the study of dreams is also linked to the role of the Church against the spread of *cle de songes*, which Guillaume Garnier underlines in the seventh chapter. In 1709, during the Enlightenment, the Roman Church condemned the *clés des songes*. The struggle against the *cle de songes* gave a new perspective: the importance of the physiology of dreams associated with the medicine of sleep. According to this perspective pastor Samuel Formey and abbot Jérome Richard proposed that dreaming is a natural phenomena and linked dreaming to sleep phenomena. During this time, the theory of fiber tension for understanding sleep phenomena was spread and thanks to this perspective Formey and Richard were able to put forward their proposal to *clés des songes*. According to this theory the imagination can not have a role in predicting the future but it can be considered only in a physiological way. Nevertheless, it seems important to analyze the other contexts, such as the Italian context, in order to better explore the important connection between the role of the Church and the role of the *clés des songes*. Therefore, more studies are needed.

This approach was also being introduced in France during the nineteenth and twentieth centuries, as Carroy shows. In fact, in the ninth chapter, Carroy mentions the main French scholars who tried to overcome the supernatural conception of dreams. This is what Pigman (2002) and Lombardo and Foschi (2008) called the “dark forest”: the huge number of scientific studies of dreams that existed before Freud but that have been neglected in contemporary literature (see Foschi et al., 2015).

Meanwhile, the clairvoyance resume the *cle de songes* linked with astrology, as Nicole Edelman described in the eighth chapter. Madame de Thèbes [Anne-Victorine Savigny (1844–1916)], a well-known clairvoyant, published *L'énigme du rêve*, translated as *explication de songes*, in 1908.

The final chapter was written by Andreas Mayer, addressing Freud. The literary translation of *Traumdeutung* seems refer to *clés de songes*. Freud considered Artemidorus to be the precursor of the psychoanalytic theory: according to the author the reason for this statement is linked to the origin of the psychoanalytic method that seems to swing between a new therapeutic method of interpretation and a hermeneutic practice derived from magic and superstition. This issue identifies the question of whether or not Freud's theory should be defined as scientific. Even if Freud quoted Artemidorus many times in his work, and considered his *Traumdeutung* as a return to the past, in contrast to those who, during this same period, used the statistical method to study dreams, he refused the symbolic reading of dreams in favor of the psychoanalytic method.

The complex issue of the relationship between “rêves” as a scientific object and “songes” as an object for interpretation must be studied in depth.

This book begins by analyzing this epistemological question, focusing on the historical course of the development of “songe.” The authors, in showing how the many translations of Artemidorus' treatise and the Byzantine and Muslim *cle de songes* differ across several periods and in several contexts according to socio-cultural change, propose the hypothesis of the continuity between oneirocriticism and *clés des songes* as interpretive practices that have persisted from Ancient time to the present. The authors follow the chronological presentation of the development of the *cle de songes* exploring the medical, prophetic, esoteric, religious, and ludic role of this art. The idea that some dreams can be signs and causes of the future has indeed moved to the border of legitimate science and *clés des songes* has been rejected in the field of popular superstitions. But oneirocriticism was simultaneously, partially rehabilitated by Freud, who wanted to develop a new practice and new knowledge which would make the meaning of dreams contingent on the past experiences of the dreamer, not on the future. The investigation of this particular corpus, which has never been done for *longue durée*, highlights differences and innovations through the ages, and also the geographical areas and cultures.

Through historicizing *clés des songes* from Ancient times to present, with the synergy between specialists from different periods, we could better understand the practices and the beliefs of different positions that have affected and still affect dreams, from Artemidorus to Freud.

For that reason the book *Clés des songes et sciences des rêves* has the merit of having reevaluated the art of *clés de songes* and its connection to scientific knowledge about dreams and their interpretation.

## Author contributions

The author confirms being the sole contributor of this work and approved it for publication.

### Conflict of interest statement

The author declares that the research was conducted in the absence of any commercial or financial relationships that could be construed as a potential conflict of interest.
